# Comparative Diagnostic Accuracy of MRI and Ultrasound in Meniscal Tear Detection: Evaluating Reliability and Limitations Against Arthroscopic Outcomes

**DOI:** 10.1155/rrp/6270476

**Published:** 2025-11-28

**Authors:** Yasser Noorelahi

**Affiliations:** Department of Radiology, Faculty of Medicine, King Abdulaziz University, Jeddah, Saudi Arabia

**Keywords:** arthroscopy, diagnosis, magnetic resonance imaging, meniscal tears, ultrasound

## Abstract

**Objective:**

Accurate meniscal tear diagnosis is essential for proper knee injury management. While MRI is the gold standard, ultrasound (US) offers a cost-effective alternative. However, its accuracy compared to arthroscopy remains uncertain. This study evaluates the diagnostic performance of US versus MRI, using arthroscopic outcomes as the benchmark for meniscal tear detection.

**Methods:**

A prospective cohort study was conducted on 208 patients aged 18–60 years with suspected meniscal injuries. Each patient underwent both US and MRI, with findings compared to arthroscopy, the gold standard for confirmation. Clinical, imaging and arthroscopic data were systematically recorded and analysed for sensitivity, specificity and diagnostic agreement. The McNemar test was used to assess differences in sensitivity and specificity between US and MRI, while the *κ* coefficient evaluated the agreement between US, MRI and arthroscopy.

**Results:**

MRI demonstrated higher diagnostic accuracy (91.83%) compared to US (84.62%) in detecting meniscal tears, using arthroscopy as the gold standard. MRI showed superior sensitivity (96.19% vs. 86.67%), specificity (87.38% vs. 82.52%) and agreement (*κ* = 0.836 vs. 0.692), with a significant difference compared to arthroscopy (*p*=0.049).

**Conclusion:**

Overall, MRI demonstrated superior diagnostic accuracy, sensitivity and specificity compared to US, suggesting that it is a more reliable imaging modality for detecting meniscal tears when arthroscopy is not feasible. In addition, US limitations in detecting complex tear patterns highlight the need for further refinement of US techniques.

## 1. Introduction

The menisci play a crucial role in maintaining knee joint homeostasis by contributing to force transmission, shock absorption, joint lubrication, stability and proprioception [1]. Meniscal tears are a common orthopaedic issue observed in both athletes and nonathletes, leading to knee pain, functional impairment and an increased risk of developing osteoarthritis [2]. Given these implications, accurate diagnosis and timely medical intervention are essential across all age groups [3]. Magnetic resonance imaging (MRI) is the preferred diagnostic modality for evaluating meniscal pathology due to its high sensitivity and specificity [4]. However, its widespread use is limited by high costs, long scanning times and contraindications in certain patient populations [4]. Ultrasound (US) has emerged as a potential alternative due to its lower cost, rapid application and ability to assess soft tissue structures, including muscles and tendons [5]. Recent advancements in US technology have improved spatial resolution, offering higher imaging detail comparable to standard musculoskeletal MRI. Consequently, US is increasingly considered a viable tool for diagnosing meniscal pathology [6]. Despite its potential, the diagnostic accuracy (DA) of US in detecting meniscal tears remains a subject of debate. Previous studies have highlighted limitations, including variations in US machine resolution and the restricted visualisation of specific meniscal regions [7].

The knee joint is highly susceptible to sports-related injuries, with meniscal tears and degenerative changes being the most frequently encountered pathologies [8]. MRI is widely recognised as the gold standard for evaluating internal derangements of the menisci due to its high accuracy and detailed soft tissue visualisation. However, it has limitations, including the inability to provide real-time dynamic imaging, limited accessibility in certain settings and high costs, which may restrict its routine use in clinical practice [9].

This study aimed to compare the diagnostic performance of US and MRI in detecting meniscal tears. Imaging findings from both modalities were correlated with arthroscopic results to determine their respective accuracies. The analysis was designed to separately assess the medial and lateral menisci, including the anterior, posterior and central segments, to provide a comprehensive evaluation.

## 2. Materials and Methods

The biomedical ethical committee approved this comparative analytical study during the period from February to October 2024. The prospective study was conducted on 208 patients aged 18–60 years with suspected meniscal injuries. The sample size was determined using a 95% confidence interval, an absolute precision of 10% and a 14% prevalence of meniscal injury, considering US's 92% sensitivity and 88% specificity for detecting meniscal tears [10]. Patients of both genders, aged 18–60 years, who required US evaluation for knee injuries, were included in the study. Those with suspected trauma-related meniscal injuries, such as pain, swelling, restricted movement or joint tenderness, were eligible if they were hemodynamically stable and had no posttraumatic fractures. Exclusion criteria included repeated knee injuries, previous medial meniscus tears, acute hemarthrosis, ligamentous instability, nontraumatic knee deformities, open wounds, critical illness, multiple traumas, loss of consciousness or diagnosed fractures. Patients who declined participation or follow-up were also excluded.

### 2.1. Data Collection

A total of 208 patients referred from the Emergency Department and Orthopaedic Department, who fulfiled the selection criteria and presented in the Department of Radiology, were enroled in the study. Demographic information, such as age, gender, duration of symptoms and presence of any clinical illness, was documented.

#### 2.1.1. US Protocol

Patients were advised to wear loose-fitting clothing for easy access to the knee. During the examination, patients were positioned supine on the examination table with the knee slightly flexed to optimise visualisation. Two-view radiographic imaging (anterior–posterior [AP] and lateral X-ray) was performed. Patients who met the inclusion criteria and had no fractures on X-ray underwent US evaluation by a radiologist. Ultrasonography has been conducted using a high-frequency linear probe (12 MHz) on a Philips Affinity 70 machine. Various knee positions and the graded compression technique were utilised for evaluating articular recesses, ensuring minimal operator bias. US examinations followed a standardised musculoskeletal scanning protocol for knee meniscus evaluation, adapted from previously published guidelines reported by Sudula et al. [11] and Akatsu et al. [12]. All scans were performed by a consultant radiologist with more than 10 years of musculoskeletal imaging experience. Since a single operator conducted all evaluations, interrater variability was not applicable.

**Figure 1:**
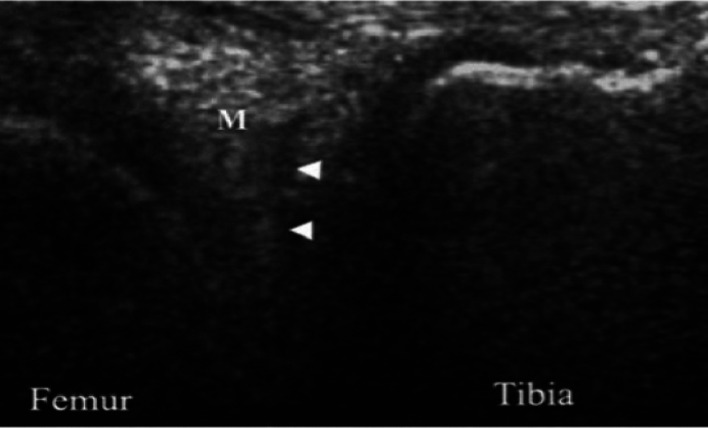
US illustration of the meniscal tear. Longitudinal ultrasound image of the posterior third of the medial meniscus (M) showing a transversely oriented linear hypoechoic tear (arrowhead).

The meniscal assessment involved evaluating echogenicity, where meniscal tears appear as hypoechoic or anechoic clefts within the echogenic meniscal structure (Figure 1). The medial and lateral menisci were examined by visualising three key components: the anterior horn, the meniscal body and the posterior horn. With the patient in a supine position, the transducer was placed along the sagittal plane, perpendicular to the longitudinal meniscal axis, to achieve optimal imaging. Various knee positions and angles were utilised as needed to enhance DA.

#### 2.1.2. MRI Protocol

MRI was conducted using a 1.5-T Toshiba MRT 1503 machine as per referral. Scans were obtained in the axial, sagittal and coronal planes, and findings were systematically recorded. During MRI, patients were positioned supine with the knee in a standardised neutral position inside a dedicated knee surface coil to optimise signal quality and spatial resolution. Standard sequences were used to evaluate different knee structures without altering limb positioning. MRI was performed following a standardised musculoskeletal protocol for knee imaging [13]. All MRI examinations were interpreted by a senior musculoskeletal radiologist with over 12 years of experience in knee MRI interpretation. As a single radiologist performed all readings, interrater reliability was not assessed.

**Figure 2:**
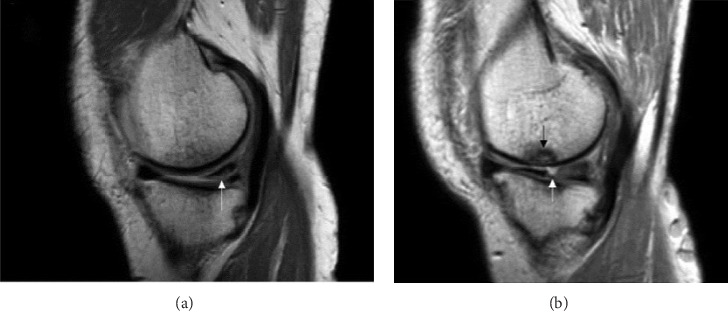
MRI illustration of meniscal tears. (a) Sagittal proton density (PD) image of the knee demonstrating an inferior surfacing tear at the posterior third of the medial meniscus (white arrow). (b) Sagittal PD image of the knee in a different patient showing truncation at the junction of the middle and posterior thirds of the medial meniscus in keeping with a radial meniscus tear (white arrow). A focal osteochondral lesion at the medial femoral condyle is also present (black arrow).

The medial and lateral menisci were visualised as wedge-shaped, hypointense structures between the femur and tibia, where meniscal tears appear as a hyperintense signal within the hypointense meniscal structure (Figure 2).

#### 2.1.3. Arthroscopy Procedure

All arthroscopic procedures were performed by experienced orthopaedic surgeons using a 30°, 4-mm-diameter arthroscope under sterile conditions with anaesthesia. A small incision allowed direct visualisation of intra-articular structures, with systematic evaluation of the medial and lateral menisci for tears, degeneration and other pathologies. Findings were recorded and compared with US and MRI to assess DA. All arthroscopies were performed by two senior orthopaedic surgeons, each with more than 15 years of surgical practice and fellowship training in sports medicine and arthroscopic knee surgery. Their extensive experience ensured diagnostic consistency and reliability of intraoperative findings. Arthroscopy was utilised as the gold standard for confirming meniscal tears. Clinical, imaging and arthroscopic data were systematically documented and analysed to assess sensitivity, specificity and diagnostic agreement.

### 2.2. Statistical Analysis

Data analysis was conducted using the Statistical Package for Social Sciences (SPSS) Version 25. Quantitative variables (age and duration of symptoms) were presented as mean ± standard deviation (SD). In contrast, qualitative variables (gender, residence, marital status, mode of injury and knee joint injury) were expressed as frequencies and percentages. Clinical, imaging and arthroscopic data were systematically recorded to assess sensitivity, specificity and diagnostic agreement. The accuracy of US and MRI was assessed using arthroscopy as the gold standard. The McNemar test was employed to compare the diagnostic performance of US and MRI by analysing differences in sensitivity and specificity. Agreement between the modalities was measured using the *κ* coefficient, with a *p* value < 0.05 considered statistically significant.

## 3. Results

The study included a total of 208 patients with a mean age of 40.24 ± 11.95 years. The majority were males (79.3%), while females accounted for 20.7% of the sample. The most common cause of injury was road traffic accidents (56.3%), followed by sports injuries (17.8%) and senile-related trauma (15.9%). Other causes contributed to 10.1% of the cases (Table 1).

### 3.1. Comparison Between US and Arthroscopy Findings in Detecting Meniscal Tears

Among 208 patients, US identified 91 true positives (86.7%), while 18 false positives (17.5%) represented cases where US detected a tear that was not confirmed by arthroscopy. Additionally, 14 false negatives (13.3%) occurred when US failed to detect a tear that was later confirmed by arthroscopy. The test correctly classified 85 true negatives (82.5%). Overall, US diagnosed 52.4% of patients with a meniscal tear, whereas arthroscopy confirmed a total of 105 cases (Table 2).

### 3.2. Comparison Between MRI and Arthroscopy Findings in Detecting Meniscal Tears

Among 208 patients, MRI accurately detected 101 true positives (96.2%), while 13 false positives (12.6%) represented cases where MRI identified a tear that was not confirmed by arthroscopy. Additionally, 4 false negatives (3.8%) were observed when MRI failed to detect a tear that was later confirmed by arthroscopy. The test correctly classified 90 true negatives (87.4%). Overall, MRI diagnosed 54.8% of patients with a meniscal tear, whereas arthroscopy confirmed a total of 105 cases (Table 3).

### 3.3. DA of US and MRI Using Arthroscopy as the Gold Standard

US exhibited a sensitivity of 86.67% and a specificity of 82.52%, reflecting its effectiveness in detecting true-positive and true-negative cases. The positive predictive value (PPV) was 83.49%, indicating that 83.49% of US-detected tears were confirmed by arthroscopy, while the negative predictive value (NPV) was 85.86%, signifying that 85.86% of negative US results were accurate. The overall DA of US was 84.62%, with a McNemar test *p* value of 0.597, indicating no statistically significant difference between US and arthroscopy (Table 4). The *κ* coefficient of 0.692 indicates a substantial level of agreement between US and arthroscopy. Compared to US, MRI demonstrated superior diagnostic performance, with a sensitivity of 96.19% and a specificity of 87.38%, highlighting its enhanced ability to detect true cases. The PPV was 95.74%, indicating that most MRI-detected tears were confirmed by arthroscopy, while the NPV was 91.83%, ensuring high reliability of negative MRI findings. Overall, MRI achieved a DA of 91.83%, surpassing that of US. The McNemar test (*p*=0.049) revealed a statistically significant difference between MRI and arthroscopy, further supporting MRI's superior diagnostic performance. The *κ* coefficient of 0.836 indicates near-perfect agreement between MRI and arthroscopy (Table 4).

## 4. Discussion

MRI is the gold standard for diagnosing meniscal tears, offering high accuracy but with cost and accessibility limitations. US is emerging as a cost-effective alternative, demonstrating comparable sensitivity and specificity. However, US remains operator-dependent and struggles with complex tear visualisation, highlighting the need for further refinement. Our study compared the diagnostic effectiveness of US and MRI in detecting meniscal tears (Figures 1 and 2). Both imaging results were cross-referenced with arthroscopic findings to determine their accuracy [14, 15]. The medial and lateral menisci, including the anterior, posterior and central sections, were analysed separately to provide a thorough evaluation. Additionally, patients were grouped by age to explore the influence of anatomical and age-related variations on US accuracy. Previous research has reported varying DA levels for US [16]. Their study found US sensitivity and specificity to be 86% and 83%, respectively. In contrast, Shetty et al. reported a similar sensitivity of 86% but a lower specificity of 69%, using an Esaote Techno machine with a 5.0–13.0-MHz linear probe [17]. However, those studies did not assess the resolution of US specifically in defining meniscal tears, making direct comparisons with our results difficult.

Our study also found that US demonstrated higher sensitivity and NPV for diagnosing medial meniscal tears, whereas specificity was higher for detecting lateral meniscal tears (Tables 2 and 4). These results align with findings from Khan et al. and Helwig et al. [14, 15]. Additionally, Wareluk and Szopinski reported similar trends, although they did not find a significant difference in the NPV between medial and lateral menisci [16]. The lower sensitivity of US in detecting lateral meniscal tears may be due to the anatomical characteristics of the lateral meniscus, particularly the proximity of the popliteus tendon, which can obscure US visualisation [11, 12, 18]. Another study reported that the anterior horn of the meniscus is more challenging to visualise on US. However, since meniscal tears are more commonly found in the posterior horn, the limited visualisation of the anterior horn does not significantly affect the overall diagnostic value of US for meniscal tear screening [19–21]. Notably, our arthroscopic findings showed no anterior horn meniscal tears. Our findings underscore the operator dependency of US, which remains a critical limitation. Factors such as radiologist training, probe positioning and anatomical challenges, particularly in the lateral meniscus, may influence DA. This variability may partly explain the differences in sensitivity and specificity compared to MRI, consistent with previous literature reported by Johnson et al. and Wareluk and Szopinski [16, 20]. Another limitation is the lack of a universally standardised US protocol for meniscal tear assessment. While MRI follows structured protocols across institutions, US techniques vary widely depending on equipment and operator preference. This heterogeneity complicates cross-study comparisons and reduces reproducibility, highlighting the urgent need for standardised, validated US guidelines.

US is a valuable screening tool, but it may not be sufficient for determining surgical intervention or predicting the exact nature of meniscal tears [10, 21]. Our study findings revealed that MRI demonstrates a higher DA (91.83%) than US (84.62%) when compared to arthroscopy as the gold standard (Tables 3 and 4). MRI showed superior sensitivity (96.19% vs. 86.67%) and specificity (87.38% vs. 82.52%), with a statistically significant difference (*p*=0.049). MRI also had a stronger agreement with arthroscopy (*κ* = 0.836) compared to US (*κ* = 0.692). This observation is consistent with previous studies and may be due to the structural differences in the lateral meniscus, particularly the presence of the popliteus tendon [13, 20, 22, 23].

MRI remains the preferred imaging method for diagnosing meniscal injuries due to its superior accuracy and ability to classify tear morphology [23, 24]. However, US has several advantages, including lower costs, real-time dynamic imaging, widespread availability and portability for bedside examinations. Nonetheless, the potential for poor-quality imaging with US remains a drawback [18].

Overall, high-resolution US demonstrated reasonable accuracy in identifying meniscal tears, particularly in the medial meniscus. However, its ability to differentiate tear patterns remains limited. Further advancements in high-resolution US probes specifically designed for knee imaging may improve their diagnostic capabilities. Future research incorporating standardised US resolution metrics may help refine its role in meniscal tear diagnosis.

## 5. Conclusion

This study emphasises the superior DA of MRI over US in detecting meniscal tears. MRI demonstrated higher sensitivity, specificity and overall agreement with arthroscopic findings, making it a more reliable imaging modality. While US remains a viable alternative, its diagnostic performance, particularly in complex tear patterns, was lower. However, US offers a cost-effective, accessible option, especially in resource-limited settings where MRI or arthroscopy may be unavailable. These findings highlight the need for further refinement of US techniques to enhance accuracy and broaden their clinical application in meniscal tear diagnosis.

## Figures and Tables

**Table 1:** Baseline information of patients.

Age in years (mean + SD)	40.24 + 11.95	

	**Frequency**	**Percent (%)**

Gender		
Male	165	79.3
Female	43	20.7
Cause of injury		
Road accidents	117	56.3
Sports injury	37	17.8
Senile-related traumas	33	15.9
Others	21	10.1

**Table 2:** Comparison between ultrasound and arthroscopy findings in detecting meniscal tear.

US findings	Arthroscopy findings		Total
	Positive	Negative	
Positive	91 (86.7%)	18 (17.5%)	109 (52.4%)
Negative	14 (13.3%)	85 (82.5%)	99 (47.6%)
Total	105	103	208

**Table 3:** Comparison between MRI and arthroscopy findings in detecting meniscal tear.

MRI findings	Arthroscopy findings		Total
	Positive	Negative	
Positive	101 (96.2%)	13 (12.6%)	114 (54.8%)
Negative	4 (3.8%)	90 (87.4%)	94 (45.2%)
Total	105	103	208

**Table 4:** Diagnostic accuracy of US and MRI using arthroscopy as the gold standard.

Diagnostic accuracy	Sensitivity (%)	Specificity (%)	PPV (%)	NPV (%)	DA (%)	*p* value	*κ* coefficient
US versus arthroscopy	86.67	82.52	83.49	85.86	84.62	0.597	0.692
MRI versus arthroscopy	96.19	87.38	88.60	95.74	91.83	0.049	0.836

Abbreviations: DA = diagnostic accuracy, NPV = negative predictive value, PPV = positive predictive value.

## Data Availability

The datasets generated during and/or analysed during the current study are available from the corresponding author (Yasser Noorelahi) on reasonable request.
